# A Western-Style Diet Influences Ingestive Behavior and Glycemic Control in a Rat Model of Roux-en-Y Gastric Bypass Surgery

**DOI:** 10.3390/jcm14082642

**Published:** 2025-04-11

**Authors:** C. Warner Hoornenborg, Edit Somogyi, Jan E. Bruggink, Christina N. Boyle, Thomas A. Lutz, Marloes Emous, André P. van Beek, Gertjan van Dijk

**Affiliations:** 1 Department of Behavioral Neuroscience, Groningen Institute for Evolutionary Life Sciences (GELIFES), University of Groningen, 9747 AG Groningen, The Netherlands; c.w.hoornenborg@rug.nl (C.W.H.); edit.somogyi33@gmail.com (E.S.); j.e.bruggink@rug.nl (J.E.B.); 2 Department of Endocrinology, University of Groningen, University Medical Center Groningen, 9713 GZ Groningen, The Netherlands; a.p.van.beek@umcg.nl; 3 School of Kinesiology, Faculty of Health Sciences, University of Western Ontario, London, ON N6G 2V4, Canada; 4 Institute of Veterinary Physiology, Vetsuisse Faculty, University of Zurich, CH-8057 Zurich, Switzerland; boyle@vetphys.uzh.ch (C.N.B.); tomlutz@vetphys.uzh.ch (T.A.L.); 5 Department of Bariatric and Metabolic Surgery, Medical Center Leeuwarden, 8934 AD Leeuwarden, The Netherlands; marloes.emous@mcl.nl

**Keywords:** Roux-en-Y gastric bypass surgery, meal patterns, glucose homeostasis

## Abstract

**Background**: Roux-en-Y gastric bypass (RYGB) surgery results in weight reduction and decreased energy intake and can ameliorate type 2 diabetes. These beneficial effects are usually attributed to changes in hunger and satiety and relatively rapid improvements in glycemic control, but these effects may depend on dietary adherence. The aim of this study is to investigate the relatively early effects of RYGB surgery on weight reduction (by focusing on eating patterns) and glycemic control in rats subjected to a healthy maintenance diet or an unhealthy Western-style diet. **Methods:** Rats were fed a high-fat diet with added sucrose (HF/S) or a low-fat (LF) diet. Body weight, high-resolution tracking of meal-related parameters, and glucose regulation after overnight fasting and during a mixed meal tolerance test (MMTT; 2 mL sweet/condensed milk) were measured before and after RYGB (RYGB+) or sham surgery (RYGB−). **Results:** HF/S feeding led to an increased body weight just before RYGB surgery, but it also caused enhanced weight loss following RYGB, which led to similar body weights in the HF/S and LF diet groups twenty-four days post-operatively. RYGB surgery and diet dependently and independently influenced meal-related parameter outcomes, where both RYGB+ and HF/S feeding resulted in shorter meal duration (*p* < 0.01), higher ingestion rates (*p* < 0.001), and increased satiety ratio (*p* < 0.05), especially in the HF/S diet group subjected to RYGB. While RYGB surgery generally improved baseline glycemic parameters including HOMA-IR (*p* < 0.01), it often interacted with diet to affect MMTT-induced hyperglycemia (*p* < 0.05), beta-cell sensitivity (*p* < 0.01), and the insulinogenic index (*p* < 0.01), with the LF rats overall maintaining better glycemic control than the HF/S-fed rats. **Conclusions:** This study shows the importance of controlling diet after RYGB surgery, as diet type significantly influences ingestive behavior, post-prandial glucose regulation, beta-cell sensitivity, and glucose tolerance after RYGB.

## 1. Introduction

Obesity is a significant public health issue, resulting in increased risk of type 2 diabetes (T2DM) [1]. Bariatric surgery has proven its success in reversing morbid obesity as well as T2DM (BMI > 35 kg/m^2^) [2,3]. In fact, bariatric surgery-induced reduction in mortality, especially in Roux-en-Y gastric bypass (RYGB), was mostly attributed to the reduction in diabetic-related death [4]. This has recently caused RYGB to be positioned as a new treatment option for obesity-related T2DM for patients with BMI < 35 kg/m^2^ [5]. Although the exact reasons for these effects are not yet fully understood, an increased production of GLP-1 seems to play a key role [6,7]. In addition to increased insulin production in beta cells, GLP-1 analogues also have effects on the brain that lead to reduced hunger and increased satiety, resulting in decreased food intake [8,9]. Furthermore, there are studies indicating changes in food preferences after bariatric surgery or treatment with GLP-1 analogues [10,11]. However, in an environment with free food choice, it is difficult to determine whether reduced food intake or changes in food preferences (and thus altered diet composition) mediate the effects of bariatric surgery. It is, therefore, important to study these effects separately.

The physiological response after a meal challenge coupled with detailed meal pattern analyses could give insight into whether diet composition is a major contributor to the outcome of RYGB surgery on the remission of T2DM. It is worth noting that recent research in meal patterns after RYGB surgery used powdered or liquid meals [12,13], leading to incomplete observations of the physiological reactions the body undergoes after a meal challenge. In addition, the type of diet may (pre) determine the settling point of body weight maintenance and may thus determine the efficacy of RYGB in weight loss and glucose homeostasis.

In the present study, we aimed to investigate the effect of RYGB in rats fed either an LF standard diet or a high-fat diet with added sucrose (HF/S) on ingestive behavioral parameters (using continued food intake registration analysis) and glucose homeostasis (using indwelling catheters for remote blood sampling) in a mixed meal tolerance test (MMTT). The findings have potential clinical implications for tailoring dietary interventions after RYGB surgery in patients, with the goal of optimizing glycemic control and preventing T2DM relapse after RYGB surgery.

## 2. Materials and Methods

### 2.1. Animals

All animal procedures were carried out in accordance with the European Community Directive (86/609/EEC) for the Care and Use of Laboratory Animals, using protocols approved and monitored by the Institutional Animal Care and Use Committee of the University of Groningen (approval code DEC7009a, approval date 2 May 2016). Adult male Wistar rats (*n* = 34; weighing 483 ± 8) were individually housed (temperature: 20 ± 2 °C, humidity: 60 ± 5%, and light/dark: 12:12 h).

### 2.2. Jugular Vein Cannulation

After acclimatization, one silicone cannula was implanted in the left jugular vein, as described before [14].

### 2.3. Diet

The LF diet was standard chow (Total energy 17.53 kJ/g; Altromin^®^ 1410, Altromin Spezialfutter GmbH & Co., Lage, Germany). The high fat with sucrose (HF/S; total energy 21.79 kJ/g) diet was made in-house by using powdered standard chow; the method was described in more detail before [15].

### 2.4. Meal Pattern Analysis

Food intake (ad libitum) was measured by an automated weighing apparatus (TSE systems, Bad Homburg, Germany, feeding sensor advanced, 259998-SEN/FED) from preoperative day-7 to post-operative day 24. The registration equipment yielded chronological data sets, with changes in hopper weights to the nearest 0.04 g, over 10 s intervals. Individual meal size (kJ), meal duration (min), meal frequency, and intermeal interval (min) were determined using meal-cluster analysis (MCA) [16] based on the “segmented package” in R (version 4.0.3) [17]. After the MCA, the ingestion rate (kJ/min; meal size divided by meal duration) and satiety ratio (min/kJ; duration of intermeal intervals per amount of food consumed in the preceding meal) were calculated.

### 2.5. Roux-en-Y Gastric Bypass Surgery

After nineteen days on their respective diets (HF/S or LF diet), the rats were divided into two groups: Roux-en-Y gastric bypass (RYGB+, *n* = 20) and sham surgery (RYGB−, *n* = 14). RYGB surgery was performed as described before [15]. In short, following isoflurane anesthesia (5% induction, 2% maintenance), the abdomens were shaved and disinfected with surgical scrub; after this, a midline laparotomy was performed. In the RYGB− group, a 7 mm gastrotomy on the anterior wall of the stomach and a 7 mm jejunotomy with subsequent closures were performed. In the RYGB+ group, (1) the proximal jejunum was divided 15 cm distal to the pylorus to create a biliopancreatic limb. After identification of the caecum, the ileum was then followed proximally to create a common channel of 25 cm. Here, a 7 mm side-to-side jejuno-jejunostomy between the biliopancreatic limb and the common channel was performed. (2) The stomach was transected 5 mm below the gastro-esophageal junction to create a gastric pouch, after which the gastric remnant was closed. (3) The alimentary limb of the jejunum was anastomosed from end to side to create a gastro-jejunostomy. After the surgical procedure, the abdominal wall was closed. Postoperative care consisted of an injection of finadyne (1 mg/L kg bodyweight) and a wet diet (HF/S or LF soaked in tap water) for five days. After this period, normal (solid) food was offered again.

### 2.6. Mixed Meal Tolerance Test

A mixed meal tolerance test (MMTT) was performed on pre-operative day-7 and on post-operative day 19 after an overnight fast (12 h). The MMTT consisted of 2 mL sweet/condensed milk (28.3 kJ; fat/sucrose ~65% of total energy) offered to the rats in a dish, which they eagerly consumed without spillage. Blood samples (200 µL in vials containing 9 µg/mL EDTA on ice) were taken via the jugular catheter at time-points −10, 1, 10, 15, 30, and 60 min before and after the start of the MMTT (at t = 0 min).

### 2.7. Glucose Homeostasis Calculations

For the glucose concentration, blood samples (50 µL) were diluted 10× (2% heparin solution) and analyzed by the ferry-cyanide method in a Technicon auto-analyzer (Technicon Instruments Corporation, Tarrytown, NY, USA) [14]. The remaining blood sample (150 µL) was centrifuged (15 min, 2500 rpm, 4 °C), and plasma was collected for insulin determination (Rat Insulin, I-Insulin Cat# RI-13K, Linco Research, Nucli Lab, The Netherlands [14]). Glucose homeostasis parameters were calculated using the equations described in Table 1.

### 2.8. Termination

**Figure 1:**
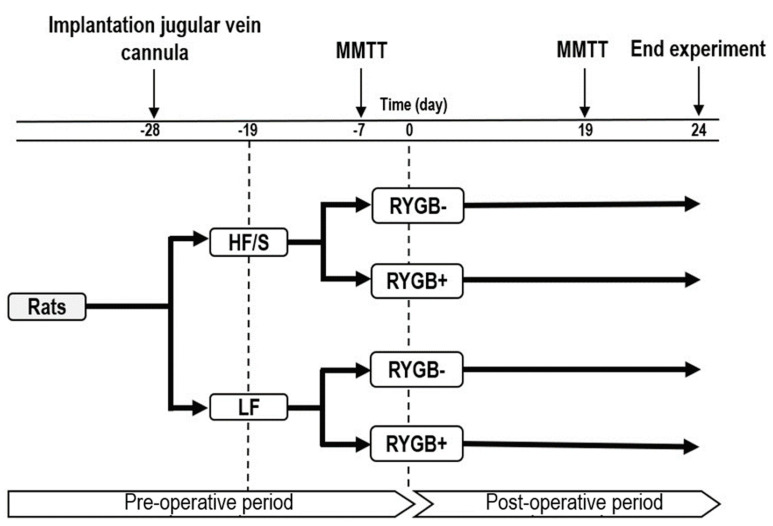
Experimental design of the experiment. Implantation of the jugular vein cannula was performed on experimental day-28. The high-fat diet with sucrose (HF/S) or the low-fat diet (LF) was fed starting on experimental day-19. The mixed meal tolerance test (MMTT) was performed on experimental days-7 and 19. The Roux-en-Y gastric bypass (RYGB+) or sham surgery (RYGB−) was performed on experimental day 0.

The rats were terminated on post-operative day 24. See Figure 1 for an overview of the experimental design.

### 2.9. Data Analysis

This study investigated the effect of surgery (RYGB+ and RYGB−) and diet (HF/S and LF) on meal-related parameters and glucose homeostasis, using a two-way ANOVA with post hoc Tukey’s pairwise multiple comparisons. Regression analysis was performed to investigate which meal-related parameter explained glucose homeostasis following RYGB surgery. The group sizes after the surgical procedures (due to surgical complication, described in detail before [15]) were as follows: RYGB+, *n* = 6 per diet group and sham surgery; LF RYGB−, *n* = 8; and HF/S RYGB+, *n =* 6. The group sizes for the post-operative MMTT were as follows: LF RYGB−, *n* = 4; LF RYGB+, *n* = 6; HF/S RYGB−, *n* = 5; and HF/S RYGB+, *n* = 6 (due to a clogged jugular vein cannula). For the purpose of this study, only the MMTT results are shown for the rats after the RYGB−/RYGB+ procedures. All data are expressed as mean ± standard error of the mean (SEM) and were analyzed using IBM SPSS software 23. *p*-values < 0.05 were considered statistically significant.

## 3. Results

### 3.1. Body Weight and Energy Intake

#### 3.1.1. Body Weight

**Figure 2:**
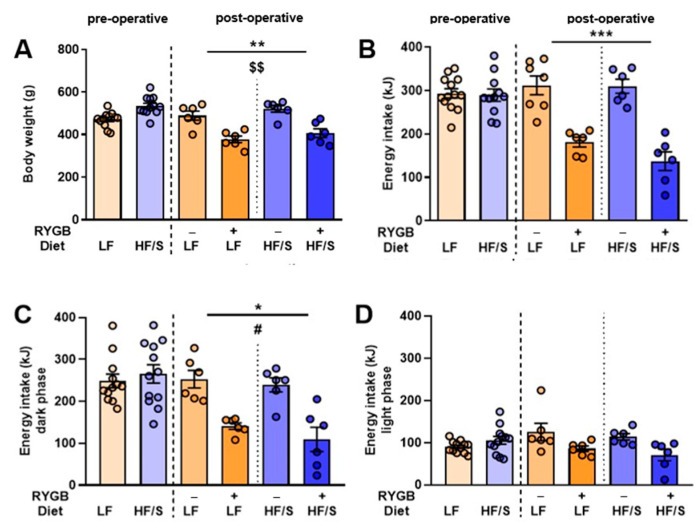
Body weight (**A**) and energy intake 24 h intake (**B**); dark phase (**C**); light phase (**D**) of rats on a high-fat diet with sucrose (HF/S) or a low-fat diet (LF) before and after Roux-en-Y gastric bypass surgery (RYGB+) or sham surgery (RYGB−). *p*-values are indicated with * (*p* < 0.05), ** (*p* < 0.01), and *** (*p* < 0.001) for surgery effects, $$ (*p* < 0.01) for diet effects, and # (*p* < 0.05) for diet × surgery effects.

Before the animals were divided into the HF/S and LF diet groups, the body weight in the HF/S (517 ± 35 g) and LF (471 ± 35 g) diet groups did not differ (*p* > 0.05). After 19 days of feeding the HF/S diet (on the day of surgery), the body weight of the HF/S group was higher compared to that of the LF diet group (*p* < 0.05; Figure 2A). Body weight decreased after RYGB+ and remained stable after RYGB−. Overall, RYGB+ resulted in a weight loss of 30% in the HF/S group and 20% in the LF group (F(1,20) = 31.007, *p* < 0.001), finally reaching comparably low levels of body weight in the HF/S RYGB+ groups relative to those subjected to LF RYGB+. A diet effect was seen on weight loss, mainly because the pre-operative body weight of the HF/S diet group was higher compared to that of the LF diet group (F(1,20) = 15.231, *p* < 0.01, Figure 2A).

#### 3.1.2. Energy Intake

One week before the surgery, no differences were seen in the energy intake between the HF/S and LF diet groups (*p* > 0.05). RYGB+ resulted in lower energy intake (Figure 2B; F(1,20) = 66.909, *p* < 0.001) compared to the RYGB− group. Energy intake during the dark phase (Figure 2C) was affected by surgery (F(1,20) = 5.706, *p* < 0.05) as well as by a diet × surgery interaction (F(1,20) = 4.736, *p* < 0.05), with the animals on the HF/S subjected to RYGB+ eating the least. No effect of diet or surgery was seen on the energy intake during the light phase (Figure 2D).

### 3.2. Meal Patterns

**Figure 3:**
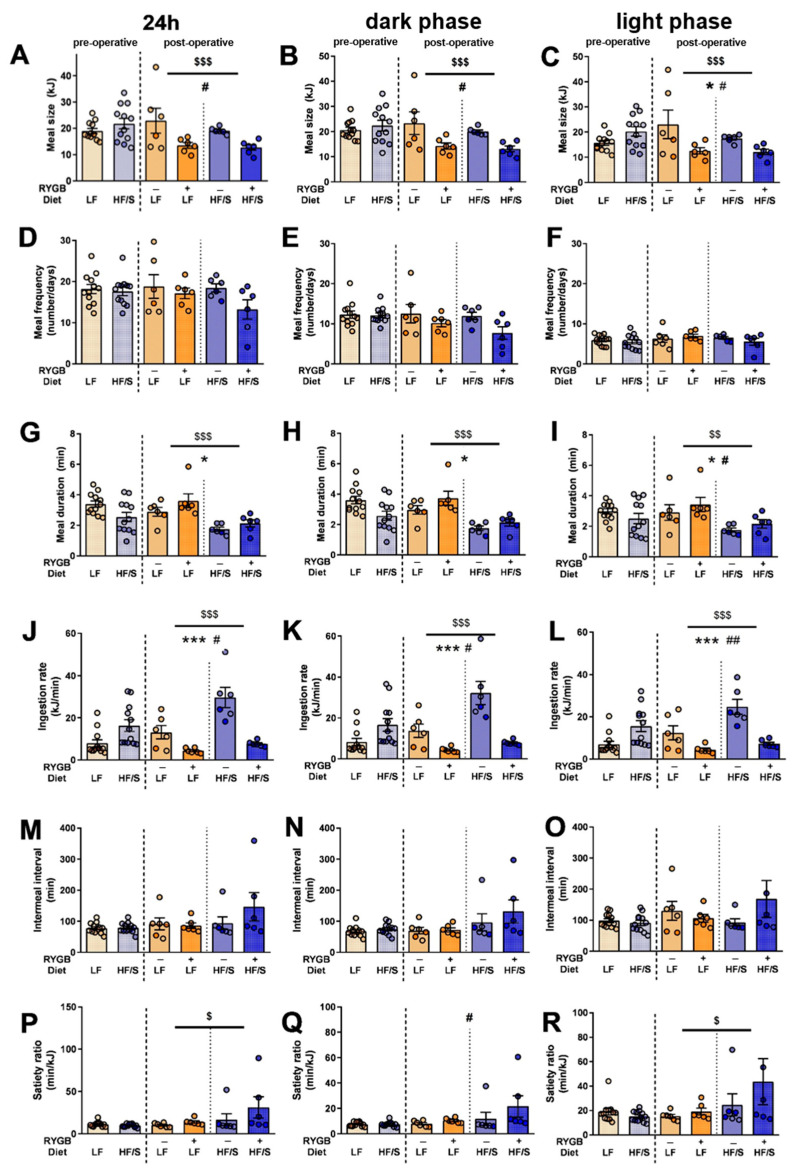
Effects of Roux-en-Y-gastric bypass surgery (RYGB+) and sham surgery (RYGB−) in rats on a high-fat/sugar diet (HF/S) or a low-fat diet (LF) on meal-related parameters over a 24 h period (**A**,**D**,**G**,**J**,**M**,**P**) and during the dark (active) phase (**B**,**E**,**H**,**K**,**N**,**Q**) and light (inactive) phase (**C**,**F**,**I**,**L**,**O**,**R**). Meal patterns are displayed as meal size (**A**–**C**), meal frequency (**D**–**F**), intermeal interval (**G**–**I**), meal duration (**J**–**L**), satiety ratio (**M**–**O**), and ingestion rate (**P**–**R**). *p*-values are indicated with * (*p* < 0.05) or *** (*p* < 0.001) for the surgery × time effect, $ (*p* < 0.05), $$ (*p* < 0.01) or $$$ (*p* < 0.001) for diet × time effects, and # (*p* < 0.05) or ## (*p* < 0.01) for the interaction of time × surgery × diet. Data on the satiety ratio and ingestion rate underwent a logarithmic transformation for the statistical analyses.

RYGB+ resulted in decreased meal size during the 24 h period (F(1,20) = 7.478, *p* < 0.001) and the dark (F(1,20) = 6.735, *p* < 0.001) and light phases (F(1,20) = 6.644, *p* < 0.001), as indicated by time × surgery interactions (Figure 3A–C). In addition, meal size was lower in the HF/S diet group compared to the LF diet group during the 24 h period (F(1,20) = 26.72, *p* < 0.001 and the dark (F(1,20) = 28.29, *p* < 0.001) and light phases (F(1,20) = 7.54, *p* < 0.001), as indicated by the time × surgery interactions. This effect was more pronounced in the HF/S RYGB+ group compared to the LF RYGB+ group during the 24 h (F(1,20) = 5.365, *p* < 0.05) and dark phases (F(1,20) = 6.170, *p* < 0.05), as indicated by a time × surgery × diet interaction. The animals did not compensate for their reduced energy intake by changing their meal frequency (Figure 3D–F). However, meal duration (Figure 3G–I) after RYGB+ was longer during the 24 h period (F(1,20) = 28.33, *p* < 0.05), dark (F(1,20) = 32.26, *p* < 0.05) and the light phase (F(1,20) = 11.44, *p* < 0.05) compared to the sham surgery. In addition, the animals on the HF/S diet had a shorter meal duration compared to the LF diet group due to a diet effect during the 24 h period (F(1,20) = 18.06, *p* < 0.001) and dark (F(1.20) = 16.42, *p* < 0.001) and light phases (F(1,20) = 11.55, *p* < 0.01). Finally, meal duration showed a time × surgery × diet interaction effect during the light phase as well (F(1,20) = 5.09, *p* < 0.05).

These effects on meal size and meal duration resulted in a decreased ingestion rate after RYGB+ during the 24 h (F(1,20) = 19.86, *p* < 0.001), dark (F(1,20) = 15.77, *p* < 0.001), and light phases (F(1,20) = 25.88, *p* < 0.001) compared to the sham surgery. The animals on the HF/S diet had a higher ingestion rate compared to the LF diet group during the 24 h period (F(1,20) = 41.56, *p* < 0.001) and the dark (F(1,20) = 33.36, *p* < 0.001) and light phases (F(1,20) = 54.27, *p* < 0.001). Overall, the decrease in ingestion rate was the highest after RYGB+ in the HF/S diet group, as indicated by a diet × surgery interaction effect during the 24 h period (F(1,20) = 7.739, *p* < 0.001) and dark (F(1,20) = 6.368, *p* < 0.001) and light phases (F(1,20) = 9.727, *p* < 0.001; Figure 3J–L). As described before, the meal frequency was not changed after RYGB, and the intermeal interval did not change either (Figure 3M–O). The satiety ratio was higher in the HF/S diet group compared to the LF diet group, as indicated by a time x diet interaction over the 24 h period (F(1,20) = 4.492, *p* < 0.05) and the light phase (F(1,20) = 5.008, *p* < 0.05). The dark (active) phase showed a time × surgery (F(1,20) = 6.688, *p* < 0.05), with a higher satiety ratio in the RYGB+ groups compared to the sham surgery (Figure 3P–R).

### 3.3. Glucose Homeostasis Parameters

#### 3.3.1. Baseline Fasted State

**Figure 4:**
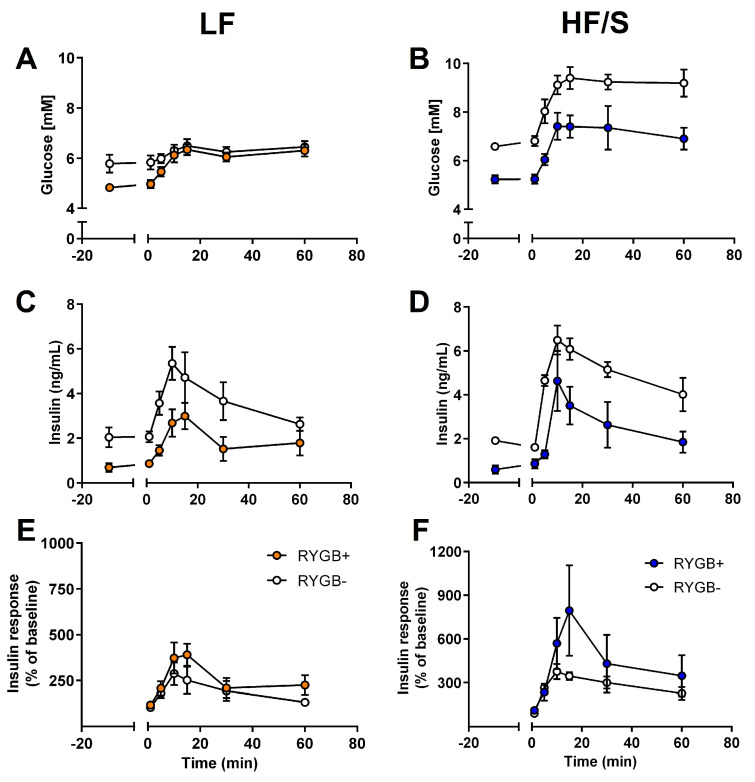
Effects of Roux-en-Y gastric bypass surgery (RYGB+) in rats fed a high-fat diet with added sucrose (HF/S) or a low-fat (LF) diet on circulating glucose levels (LF RYGB+/− panel (**A**); HF/S RYGB+/− panel (**B**)), circulating insulin levels (LF RYGB+/− panel (**C**); HF/S RYGB+/− panel (**D**)), and the relative insulin response (as % of baseline; LF RYGB+/− panel (**E**); HF/S RYGB+/− panel (**F**)) during a mixed meal tolerance test (MMTT).

Blood glucose levels in the fasted state (i.e., before the start of the MMTT) were affected by surgery (F(1,17) = 35.68, *p* < 0.01) and diet (F(1,17) = 9.819, *p* < 0.01), and insulin levels were affected by surgery alone (F(1,17) = 26.55, *p* < 0.01). In short, the RYGB− rats had higher circulating glucose and insulin levels compared to those in the RYGB+ groups (Figure 4 and Table 2). Mathematical determinants of glucose homeostasis parameters during the fasted state (Table 2) showed a decreased insulin resistance in the RYGB+ group (as indicated by a surgery effect on HOMA-IR; F(1,17) = 39.496, *p* < 0.01), and lower beta-cell sensitivity (as calculated by HOMA-B) was found in the HF/S-fed rats compared to the LF-fed rats (F(1,17) = 6.006, *p* < 0.05).

#### 3.3.2. Post-Prandial State

During the MMTT, the two-way ANOVA showed that AUC_gluc_ (Table 2) was affected by diet (F(1,17) = 30.844, *p* < 0.0001), surgery (F(1,17) = 17.940, *p* < 0.01), and an interaction of diet × surgery (F(1,17) = 6.867, *p* < 0.05). The interaction is explained to indicate that AUC_gluc_ increased by RYGB+ in the LF diet group, while such an effect was not observed in the HF/S-fed rats after RYGB+. Peak levels of blood glucose were usually found at t = 15 min following the start of the MMTT and were affected by diet (F(1,17) = 25.080, *p* < 0.001), surgery (F(1,17) = 7.368, *p* = 0.015), and an interaction of diet × surgery (F(1,17) = 5.427, *p* = 0.032).

The AUC_ins_ was affected by surgery (F(1,17) = 17.879, *p* < 0.001), with significantly lower levels in the RYGB+ rats relative to the RYGB− rats. No effects were observed on the relative insulin responses (Figure 4E,F), although these tended to be increased in the HF/S rats subjected to RYGB+. Beta-cell sensitivity (based on AUC_ins_/AUC_gluc_, Table 2) was influenced by surgery (F(1,17) = 8.284, *p* < 0.01), with generally lower levels found in the RYGB+ rats. Beta-cell sensitivity was also affected by an interaction between surgery and diet (F(1,17) = 6.682, *p* < 0.01), with a profound reduction following RYGB+ in the LF rats, while the AUC_ins_/AUC_gluc_ was more comparable in RYGB+/− in the HF/S diet group.

The early insulin secretion (calculated by the insulinogenic index_0–10_, Table 2) was affected by surgery (F(1,17) = 20.47, *p* < 0.01), diet (F1,17) = 30.78, *p* 0.01), and a diet × surgery interaction (F(1,17) = 16.22, *p* < 0.01). Overall, the drop in the insulinogenic index_0–10_ from RYGB− to RYGB+ was the largest in the LF rats. A surgery effect remained in the insulinogenic index_0–15_ (F(1,17) = 6.318, *p* < 0.05), with lower levels in the HF/S groups compared to the LF diet group.

### 3.4. Influence of Meal Pattern Parameters on Glucose Homeostasis

Feeding behavior is tightly connected to the regulation of glucose homeostasis [20]. For that reason, we performed a regression analysis with “AUC_gluc_”, “AUC_ins_”, and “AUC_ins_/AUCg_luc_” as dependent factors and the meal-related parameters, “meal size”, “meal duration”, “meal frequency”, and “ingestion rate”, as independent factors. Firstly, a negative correlation between meal size (kJ) and AUC_gluc_ was seen after RYGB surgery irrespective of diet type (B = −14.98, R^2^ = 0.445, *p* = 0.0250). When separating diet conditions, this negative correlation after RYGB surgery was only seen in the HF/S-fed rats (B = −27.03; R^2^ = 0.9011; *p* = 0.0136). Second, a negative correlation between meal frequency and AUC_gluc_ was seen after RYGB surgery (B = −9.601; R^2^ = 0.6044; *p* = 0.049), with again only the HF/S diet group contributing the most to this effect (B = −12.06, R^2^ = 0.8811, *p* = 0.0181). The same was found for meal frequency in relation to AUC_ins_/AUC_gluc_ in the HF/S diet group only (B = 1.361; R^2^ = 0.7028; *p* = 0.0403). No correlation between meal duration and the post-prandial glucose homeostasis indices was found (AUC_gluc_, AUC_ins_, AUC_ins_/AUC_gluc_).

## 4. Discussion

In the present study, a high-fat diet with added sucrose (HF/S) contributed to an increased body weight just before Roux-en-Y gastric bypass (RYGB) surgery, but also enhanced weight loss following RYGB, which finally led to similar body weights in the HF/S and LF diet groups 3 weeks after RYGB. The enhanced reduction in energy intake during the dark (active) phase in the HF/S diet group after RYGB+ likely contributed to this enhanced weight loss [15]. Previously, we reported that the lower intake in the RYGB+ HF/S group was not associated with signs of enhanced surgery-induced malaise, as witnessed by the less severely affected locomotor activity and body temperature rhythmicity in the HF/S rats relative to those observed in the LF-fed rats [15]. High-resolution tracking of meal-related intake parameters in the present study revealed that relative to the sham-operated controls, RGYB caused lower energy intake particularly by reducing meal size irrespective of diet, without significant changes in the intermeal interval and meal frequency (i.e., likely ruling out non-specific effects such as sickness/malaise [21]).

In more detail, the major finding of the meal pattern analysis is that meal size decreased to 50% of the preoperative meal after RYGB in both diet groups, with animals needing more time to eat these smaller meals. Because the intermeal interval and meal frequency did not change by RYGB, the reduced meal size as the result of RYGB was apparently not compensated for by eating more frequent meals, which is an indication of reduced intake by increased meal-related satiety [22]. RYGB also caused the rats to have slower eating rates during meals compared to the sham animals. Finally, the HF/S-fed animals subjected to RYGB showed an increased satiety ratio during the 24 h and light (inactive) period; these results are consistent with those of other rodents [22] and clinical studies [23,24]. The combination of surgery (RYGB+) and diet (HF/S) could have triggered a stronger ileal brake [25] relative to the LF diet, leading to a higher satiety ratio and decreased energy intake. Previous studies showed that rats avoided a calorically dense diet during a food preference test after bariatric surgery (RYGB and vertical sleeve gastrectomy), and increased the intake of their “regular” food after the food preference test to compensate for the weight loss [26,27]. It may be speculated that in our study, the HF/S RYGB+ group could not compensate for their weight loss because we “forced” the rats to stay on the HF/S diet after surgery, resulting in a difference in weight loss compared to the LF RYGB+ group. Whether this effect on energy intake and meal-related parameters was due to a reduced liking of the diet, as seen in other studies [28,29], or due to energy malabsorption [30] cannot be concluded based on our data. However, eating fast (especially seen in the HF/S-fed animals) is indicative of malabsorption [31] and shows similarities with binge eating, despite the fact that overall energy intake during a meal is similar in both diet groups [32].

To be able to compare glycemic responses, we used sweet and condensed milk in the mixed meal tolerance test (MMTT), which contained approximately the same energy as a regular meal (i.e., calculated from the meal pattern analysis in the present study). From the viewpoint of nutrient composition, this standardized MMTT was different from both diets; however, it was more comparable to the HF/S diet than the LF diet. The mixed meal was offered to a rat in a dish, from which the content was avidly ingested without spillage, and blood samples were taken remotely from an indwelling catheter that was exteriorized outside the cage impacting the rats as little as possible during the MMTT. To our knowledge, such a “stress-limiting” MMTT approach in rats that underwent RYGB has not been employed before and gives, in our opinion, a more realistic view regarding glucose homeostasis. While RYGB+ surgery was mostly effective in improving baseline fasting glucose and insulin parameters (including HOMA-IR, but not for HOMA-B), the effects of RYGB surgery on glycemic control during the MMTT often interacted with diet. For example, a significantly higher MMTT-induced elevation in AUC_gluc_ was observed by RYGB+ in the LF-fed rats; however, AUC_gluc_ in the HF/S-fed RYGB + and RYGB− rats appeared similar. Despite this surgery effect on AUC_gluc_ in the LF diet group, the absolute peak blood glucose levels (usually happening 15 min after the start of the MMTT) were indistinguishable in the LF RYGB and sham operated controls, indicating similar levels of MMTT-related glucose tolerance. In the HF/S condition, however, the MMTT-induced absolute peak blood glucose levels were significantly lower in the RYGB+ rats relative to the sham operated controls, indicating improved MMTT-related glucose tolerance by RYGB+ in the HF/S condition. The MMTT-induced AUC_gluc_, as well as absolute peak levels, were nevertheless still significantly higher in the HF/S-fed rats relative to the LF-fed rats irrespective of surgery, indicating that the HF/S-fed rats were overall less tolerant than the LF-fed rats.

The MMTT-induced insulin excursions (i.e., assessed by AUC_ins_) were higher in the HF/S-fed rats relative to the LF rats irrespective of surgery; however, the MMTT-induced peak insulin levels did not attain significant differences between the diet and surgery groups. The calculated beta-cell function and insulinogenic indices were overall highest in the LF RYGB− group and dropped markedly by RYGB+. In the HF/S group, however, these indices were already lowered significantly in the RYGB− control group relative to the RYGB− LF control group, and they did not decline further in the RYGB+ rats relative to the RYGB− rats in the HF/S diet group.

An explanation for these results is that RYGB+ in the present study resulted in glucose disposal dysfunction due to an insufficient adaptation of beta-cell function [2]. This is particularly seen in the rats fed the LF diet after RYGB+, while the rats fed the HF/S diet may have already had glucose disposal dysfunctions, per sé (i.e., irrespective of RYGB surgery), in the context of this MMTT. Despite the calculated reduction in beta-cell function and the insulinogenic index, it should be noted again that the MMTT-related glucose tolerance in the LF diet group was not affected by surgery, while in the HF/S diet group, the MMTT-related glucose tolerance was improved by RYGB+ (i.e., judged from the difference in peak circulating glucose level in RYGB+ and sham controls in the HF/S condition, but not in the LF diet condition). Because of the unfamiliarity of the LF-fed rats with fat and sugar loads, this might, to some extent, explain the worsening of the insulinogenic index after RYGB in the rats fed the LF in contrast to the rats fed the HF/S diet. The long-term health consequences of differences in fuel excursions in the LF and HF/S rats remain to be investigated. For example, future studies could investigate whether the health consequences of RYGB differentially improve over time in LF versus HF/S feeding rats or whether the different diets alter the risk for early or late phase dumping after RYGB [33]. These fuel excursions may be related to ingestive behavior, as the animals in the HF/S RYGB+ group showed a negative correlation between meal size and AUC_gluc_ and meal size and beta-cell function (AUC_ins_/AUC_gluc_), which may indicate that those that ate the smallest meals had the most difficulties in processing the glucose influx associated with the MMTT (i.e., in this case, eating ensure).

As mentioned already above, the interaction effects of surgery and diet on post-prandial glycemic control are in contrast with the findings in the fasted condition right before the MMTT, where glycemic control in the fasted state was mainly improved by RYGB+ surgery. In this study, the RYGB+ decreased glucose and insulin concentrations in the fasted state could be attributed to reduced adipose tissue [15], which could in turn have improved insulin sensitivity (as evidenced by the lowering in HOMA-IR [34]). This effect is consistent with other rodents [35,36] and clinical findings [37,38].

Although we did not measure gut hormones in our study, we acknowledge that they might have played a role in the observed results [20]. In general, the positive impact of RYGB surgery on glucose homeostasis is often linked to an increased secretion of GLP-1 [39], and it also has been demonstrated that GLP-1, together with peptide YY, contributes to weight loss after RYGB [40]. While RYGB in the present study clearly induced weight loss, it did not improve glycemic control on all fronts, and it would be of interest to relate those results to circulating gut hormones or other endocrine/autonomic factors. Additionally, the gut microbiota could have influenced the modifications in glucose regulation and eating behavior as well, as RYGB can alter the composition of the gut microbiota as early as 1 week after RYGB surgery, independently of weight or diet [41]. Therefore, it is of interest to study diet effects on RYGB-induced weight loss and glucose homeostasis over an extended time frame to compare early (like in this study) and later RYGB-induced alterations in glucose homeostasis linked to the secretion of gut hormones. This can be completed in a cross-over design (i.e., from HF/S to LF feeding and v.v.) with pair-fed controls and the addition of groups, where the gut microbiota is transplanted from animals on an HF/S diet to an LF diet (and v.v.) after RYGB surgery.

Finally, the results from the regression analysis indicated that animals that did not control their meals after RYGB (i.e., eating relatively small meals fast) had more disturbed glucose homeostasis. Overall, these data emphasize the importance of tailoring dietary interventions and the need to adhere to dietary guidelines after RYGB for human patients to optimize glycemic control and prevent T2DM relapse.

## 5. Conclusions

Our study highlights that diet composition plays a significant role in the effects of Roux-en-Y gastric bypass surgery on ingestive behavior and glycemic control. While a diet high in fat and sugar may induce an increased level of satiety following RYGB, it could exacerbate hyperglycemic episodes, leading to poor glycemic control in patients indicative of insufficient beta-cell adaptation, potentially obstructing recovery from T2DM. Regular glucose monitoring (e.g., by using a subcutaneous continuous glucose sensor) and dietary counseling may help mitigate these risks. In addition, further clinical research based on our findings can validate the specific dietary thresholds for hyperglycemia prevention.

## Figures and Tables

**Table 1:** Relevant equations for blood glucose homeostasis parameters. Fasting glucose homeostasis parameters: HOMA-IR represents the insulin-mediated inhibition of hepatic glucose production, where lower values are better. HOMA-B is an index of insulin secretory function. Post-prandial glucose homeostasis parameters: the incremental (i.e., above baseline) area under the curve (AUC) of glucose and insulin are calculated using trapezoidal integration. The intervals $x_{n}-x_{n-1}\;$ are divided into n equal subintervals: $h=\frac{x_{n}-x_{n-1}\;}{n}$. The ratio of the AUC of the circulating insulin and glucose responses represents the ability of insulin levels to decrease circulating glucose levels and is seen as an index for glucose-mediated beta-cell function [14,18,19]. Finally, the insulinogenic index represents early insulin secretion during the corresponding time period [19].

Fasting glucose homeostasis parameters		
1.	HOMA-IR	$HOMA-IR=\frac{{\left[insulin\right]}_{0}\cdot {\left[gluocse\right]}_{0}}{22.5}$
2.	HOMA-B	$HOMA-B=\frac{{20\;\cdot \;[insulin]}_{0}}{{\left[glucose\right]}_{0}-63}$
Post-prandial glucose homeostasis parameters		
3.	AUC_ins_, AUC_gluc_	$Area=\int_{a}^{b}ydx\;\approx \;\frac{1}{2}h\;[y_{0}+2\left(y_{0}+y_{0}+\ldots +y_{n-1}\right)+y_{n}]\;\;$
4.	Beta-cell sensitivity	$Beta\;cell\;sensitivity=\frac{{AUC}_{insulin}}{{AUC}_{glucose}}$
5.	Insulinogenic index	$Insulinogenic\;index\;(x_{0}-x_{n})=\frac{{\left[insulin\right]}_{x}-{\left[insulin\;\right]}_{0}}{{\left[glucose\right]}_{x}-{\left[gluocse\right]}_{0}}$

**Table 2:** The effect of Roux-en-Y gastric bypass (RYGB+) and sham (RYGB−) surgery on baseline glucose and insulin levels and mathematical indices of insulin sensitivity and resistance. Insulin indices were calculated via HOMA-IR and the insulinogenic index (0–5, 0–10, 0–15), and beta-cell sensitivity was calculated via HOMA-B and AUC_ins_/AUC_gluc_. Effects of surgery (s), diet (d), and their interaction (d × s) are indicated as abbreviated capitals (*p* < 0.01) or lowercase letters (*p* < 0.05). Data on incremental AUC_gluc_ underwent a logarithmic transformation to reach normal distribution (for the statistical analyses).

	LF RYGB−	LF RYGB+	HF/S RYGB−	HF/S RYGB+	Main Effect
Fasting glucose homeostasis parameters					
Glucose mM	5.80 ± 0.32	5.26 ± 0.20	6.70 ± 0.16	4.84 ± 0.18	S, D
Insulin (ng/mL)	2.06 ± 0.34	0.77 ± 0.14	1.76 ± 0.09	0.74 ± 0.19	S
HOMA-IR	0.543 ± 0.119	0.165 ± 0.028	0.527 ± 0.038	0.171 ± 0.050	S
HOMA-B	0.99 ± 0.05	0.69 ± 0.13	0.42 ± 0.13	0.60 ± 0.16	D
Post-prandial glucose homeostasis parameters					
AUC_gluc_	29.9 ± 7.5	70.2 ± 8.7	139.9 ± 19.2	108.7 ± 31.0	D, S, D × S
AUC_ins_	94.6 ± 40.8	67.1 ± 23.9	189.8 ± 11.4	109.5 ± 26.5	D, s = 0.058
AUC_ins_/AUC_gluc_	2.9 ± 0.7	0.9 ± 0.3	1.5 ± 0.2	1.2 ± 0.2	S, d × s
Insulinogenic index 0–5 min	2.89 ± 2.82	1.22 ± 0.32	4.47 ± 1.44	0.52 ± 0.23	
Insulinogenic index 0–10 min	7.88 ± 0.86	1.43 ± 0.34	2.43 ± 0.66	1.79 ± 0.56	D, S, D × S
Insulinogenic index 0–15 min	4.00 ± 1.09	1.46 ± 0.27	1.79 ± 0.14	1.42 ± 0.40	S

## Data Availability

The original contributions presented in this study are included in this article. Further inquiries can be directed to the corresponding authors.

## Abbreviations

The following abbreviations are used in this manuscript:

AUC	area under the curve
BMI	body mass index
GLP-1	glucagon-like peptide-1
HF/S	high fat with added sucrose
LF	low fat
MCA	meal cluster analysis
MMTT	mixed meal tolerance test
RYGB	Roux-en-Y gastric bypass
T2DM	type 2 diabetes mellitus
